# Primary intra- and extradural extramedullary mesenchymal chondrosarcoma with isolated punctate calcification: case report and literature review

**DOI:** 10.1186/s12883-022-02645-x

**Published:** 2022-03-23

**Authors:** Meigui Chen, Qingquan Lai

**Affiliations:** Department of Radiology, The Second Affiliated Hospital of Fujian Medical University, 34 N, Zhong-Shan-Bei Street, Quanzhou City, Fujian Province China

**Keywords:** Case report, Mesenchymal chondrosarcoma, intra- and extradural, Dumbbell-shape, Calcification

## Abstract

**Background:**

Mesenchymal chondrosarcoma (MCS) is an ultra-rare, high-grade subtype of chondrosarcoma affecting both bone and soft tissues. Extra-skeletal MCS rarely occurs in intra- and extradural regions.

**Case presentation:**

We presented a case of intraspinal dumbbell-shaped MCS at the T12-L2 level with isolated punctate calcification in a 19-year-old male complaining of progressive lower back pain. Surgical treatment for removal of the tumor was performed. The postoperative pathological result confirmed MCS. The patient showed symptomatic improvement and follow-up MRI showed no evidence of recurrence or metastasis for nearly 1 year after surgery.

**Conclusions:**

CT and MRI play an important role in differential diagnosis for intraspinal MCS. MCS should be added to the differential diagnosis of intraspinal dumbbell-shaped tumors, especially when radiological examinations reveal punctate calcification in a homogeneous enhanced tumor without dural tail sign. However, the final diagnosis depends on histopathological results. Despite the good prognosis of intraspinal MCS, close follow-up after operation is still necessary.

## Background

Mesenchymal chondrosarcoma (MCS) is an unusual variant of chondrosarcoma affecting both bone and soft tissues. Extra-skeletal MCS most commonly occurs in the brain, and much rarer in intra- and extradural regions. We reported a case of primary intra- and extradural extramedullary MCS at the T12-L2 level in a teenager and discussed its clinical and radiological features after reviewing pertinent literature, and aimed to identify radiological characteristics to distinguish MCS from other intraspinal tumors.

## Case presentation

A 19-year-old male complaining of progressive lower back pain with a 2 weeks history was admitted to our hospital in November 2020. Physical and neurological examinations showed hypoesthesia below the dermatome of L1, hyporeflexia of the patellar tendon reflex bilaterally. Bladder and bowel dysfunction were also present. As the clinical manifestation was compatible with conus medullaris syndrome, a compressive lesion to spinal cord at the lumbar spine was suspected.

**Fig. 1:**
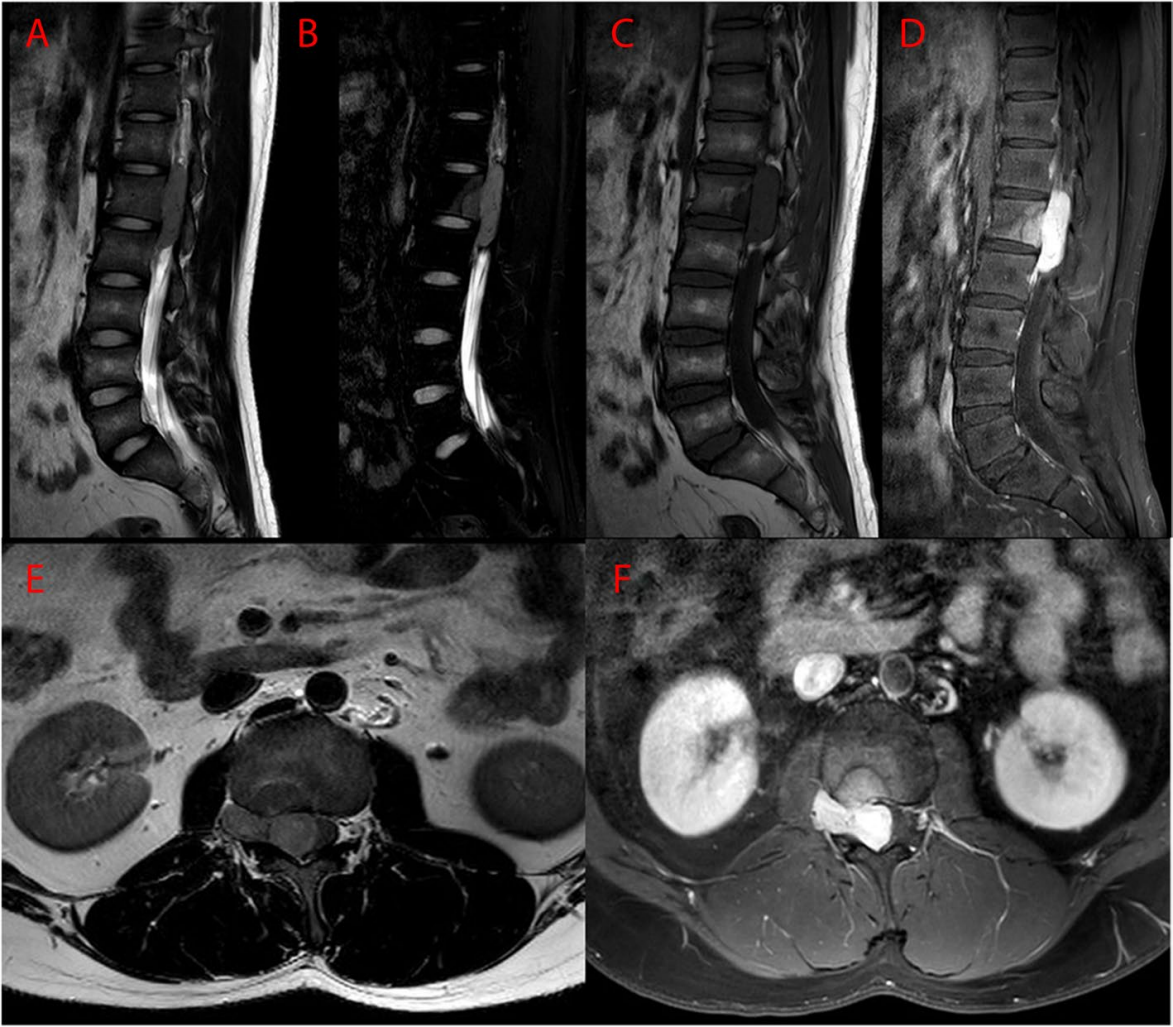
Preoperative MR images. **A** Sagittal T2WI, **B** Sagittal T2WI with fat suppression, **C** Sagittal T1WI, **D** Sagittal T1WI with gadolinium enhancement, **E** Axial T1WI, **F** Axial T1WI with gadolinium enhancement. Dumbbell tumor could be visualized at the L1 level

**Fig. 2:**
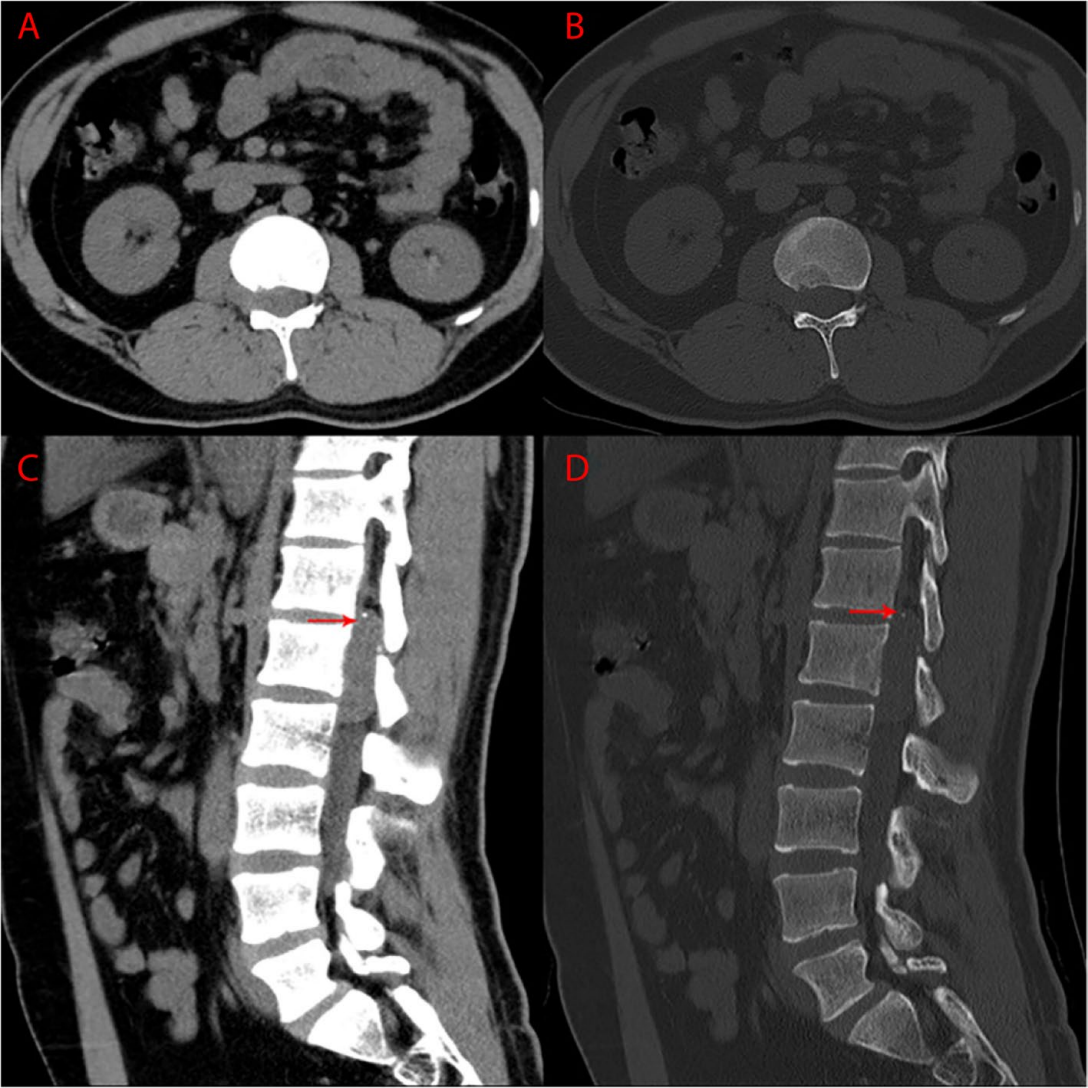
Preoperative CT images. **A**, **B** Axial images. **C**, **D** Sagittal images. CT images revealed isolated punctate calcification in the mass (arrows)

Lumbar spinal MRI (Fig. 1) showed an intraspinal dumbbell-shaped soft tissue mass measuring 3.4 × 4.9 × 0.7 cm at the level of T12-L2 vertebra, which extended from central canal to the extraforminal space. The tumor pushed the dural sac to the left side, resulting in conus medullaris compressed. The tumor appeared isointense on T1-weighted images (T1WI) and mildly hyperintense on fat-suppression T2-weighted images (T2WI) to the normal spinal cord. After intravenous gadolinium administration, the tumor presented pronounced and homogeneous enhancement on T1WI without thickening and enhancement of adjacent dura (the dural tail sign). The tumor was well-defined, and there was no obvious evidence for bony destruction or pathological fracture in T12-L2 vertebra. However, focal heterogeneous signal intensities could be seen in posterior part of L1 vertebral body. Radiologists thought that these abnormal signals might represent bone marrow edema, inflammation and ischemia as a result of tumor compression, although they could not completely exclude the possibility of tumor invasion. CT images (Fig. 2) revealed isolated punctate calcification in the tumor, decreased bone density of posterior part of L1 vertebral body, and focal bone defect in posterior edge of L1 vertebral body. Based on these image findings, radiologists firstly thought the tumor to be a neurogenic tumor or meningioma.

Surgical treatment with tumor resection and transvertebral pedicle screws internal fixation in T12 - L2 was performed. During the operation, the dumbbell-shaped tumor was found in intra- and extradural space. Grossly, the tumor sized 4.0 × 4.0 × 2.0 cm, and was grayish purple, well-encapsulated, firm, hypervascular. Careful examination revealed that the tumor was not derived from any nerve root and not adherent to the cord. The tumor was attached to the inner surface of dura mater at the L1 root sleeve, flowing along the L1 nerve root to the extradural space. According to these findings, the tumor was highly suspected as meningioma.

**Fig. 3:**
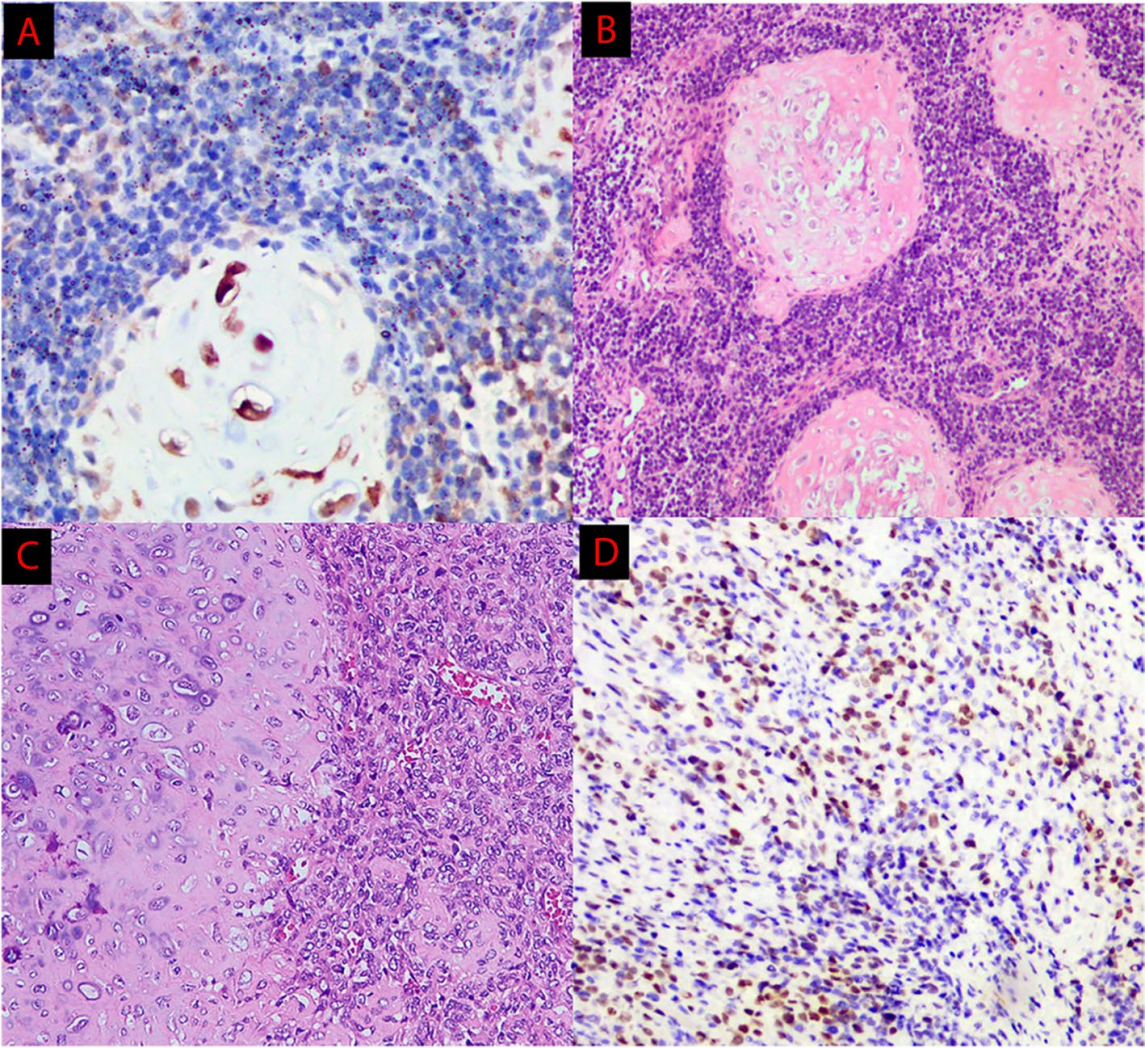
Postoperative histopathological findings. **A** The chondroid component stained positive for S-100 protein. **B** Round or spindle-shaped undifferentiated cells with island-like regions of cartilage **C** A biphasic pattern. **D** spindle-shaped undifferentiated cells stained positive for myogenin

The postoperative histological examinations (Fig. 3) revealed that the tumor exhibited a biphasic pattern, composing of undifferentiated mesenchymal cells intermixed with islands of hyaline cartilage. The primitive mesenchymal cells were round, oval, or spindle-shaped with a high nucleocytoplasmic ratio. As for immunohistochemistry, the tumor cells were positive for CD99, SMA, BCL-2, STAT6, with 20% Ki-67 positive nuclei, while negative for DES, S-100, SOX10, CD117, P63, CK, CD34. Collectively, the tumor was diagnosed with mesenchymal chondrosarcoma.

The patient’s symptoms gradually disappeared, and no any neurological deterioration happened after surgery. Although metal artifacts caused by pedicle screws distorted the quality of images, the tumor seemed to have been completely resected based on postoperative CT and MRI. The patient was followed as an outpatient and underwent MRI every 4 months. Since most MCS was malignant tumors that originated in the bone, the changes of abnormal signal of the L1 vertebral body might be important and worthy to be described. However, metal artifacts made the abnormal signal unidentifiable, we couldn’t clearly describe these findings in follow-up MRI. The last follow-up was performed in August 2021. The patient described no clinical symptoms, and there was no evidence of recurrence or metastasis. The overall follow-up period was nearly 1 year.

## Discussion and conclusions

Although the first case of extra-skeletal MCS was reported by Dowling in 1964, a primary intraspinal dumbbell-shaped MCS with isolated punctate calcification is a rare phenomenon. To the best of our knowledge, seven cases of intraspinal dumbbell-shaped MCS have been reported in the English literature since 1978. The eight cases of intraspinal dumbbell-shaped MCS including the current case were summarized in Table 1 [1–7].

**Table 1:** Literature review of primary spinal dumbbell-shaped MCS published from 1978 to 2019

Case report	Age	Sex	Location	Calcification	Dural attachment	Dural tail sign	Treatment	Outcome
Chan et al. 1984 [1]	10	F	T3–4 (ED)	–	+	N/A	TR/RT/CT	Alive, 18 months
Reif et al. 1987 [2]	3	M	L1–5 (I&ED)	–	+	N/A	RT/CT	Died, metastasis
Di et al. 1989 [3]	40	F	L5–S1 (ED)	–	+	N/A	TR/RT/CT	Alive, 5 years
Rushing et al. 1996 [4]	48	F	L5–S1 (ED)	–	+	N/A	GTR/RT	Died, metastasis
Bae et al. 2011 [5]	25	M	T7 (I&ED)	+ massive	N/A	–	TR/RT/CT	Alive, 2 years
lida et al. 2014 [6]	10	F	L4 (I&ED)	–	N/A	–	TR	Alive, 3 years
Chen et al. 2016 [7]	26	F	L3–5 (ED)	+ massive	+	–	TR/RT	Alive, recurrence
Current case	19	M	T12-L2(I&ED)	+ punctate	+	–	TR	Alive, 1 years

Age (years); *CT* Chemotherapy, *ED* Extradural, *F* Female, *GTR* Gross total resection, *I&ED* Intra- and extradural, *M* Male, *N/A* Limited information, *RT* Radiotherapy, *TR* Total resection

Most of these patients were under 30 years of age, with a mean age of 22.6 years. Generally speaking, adult-onset intraspinal extramedullary tumors are mainly meningioma or schwannoma; however, MCS should also be taken into consideration in the differential diagnosis. To date, an increasing number of reports suggest that the incidence of MCS is associated with age and approximately 70% of the cases occurring during the second and the third decades of life.

MRI and CT are the imaging modality of choice for intraspinal tumors. Radiographs typically give MCS an appearance of soft tissue mass or osteolytic, ill-defined lesion, or well-defined borders with sclerosis. Since an admixture of small cells with cartilaginous tissue results in low water content, MRI signal characteristics of intraspinal MCS are typical with isointense signals on T1WI while heterogeneously high intensity or isointensity on T2WI. The overall signal intensity is homogeneous, but if there is calcification, it appears as various signals in MRI, depending on the amount and distribution of low T1/T2 signal calcification. Both calcified and noncalcified areas can be clearly showed on T2WI, while enhanced MR images typically reveal the heterogeneous enhancement of both regions. On CT images, intraspinal MCS has attenuation similar to muscle with different degree calcification [8]. The current case was quite consistent with the common situations. However, due to the unreliability of imaging examinations to identify MCS, clinicians should also consider the schwannoma, meningioma, hemangioblastoma, neurofibroma while making a diagnosis.

The term “dumbbell tumors” does not refer to the dumbbell shape, but it is a conceptual term meaning tumors that connect two or more separate regions, such as intradural, epidural, and paravertebral spaces. Therefore, among the seven previously reported cases, three cases with intra- and extradural space involvement were thus considered to be dumbbell tumors. The differential diagnosis of spinal dumbbell tumors includes a broad spectrum of pathological entities, such as neurogenic neoplasm, meningioma, vascular lesion, hematopoietic neoplasm, etc. [9]. It is certainly a diagnostic challenge to differentiate dumbbell-shaped tumors on MRI. Intraspinal MCS, although a rare entity, must be added to the differential diagnosis of dumbbell-shaped spinal tumors. Dumbbell-shaped MCS is often misdiagnosed as schwannoma, since 90 % of spinal dumbbell tumors are schwannoma. Schwannoma is frequently associated with hemorrhage, intrinsic vascular changes, cyst formation and fatty degeneration. Therefore, MRI of spinal schwannoma typically reveals a well-defined nodular intraspinal mass with inhomogeneous enhancement or ringlike enhancement. In addition, schwannoma should be first suggested when a thickened enhancing adjacent nerve root is found.

Among the seven previously reported cases, five cases showed dural attachment, and none had the dural tail sign, similar to the current case. Thus, in our case, we believed that the tumor might have arisen from embryonic rest cells of cartilage within the dura at the root sleeve and extended intradurally and extradurally to form a dumbbell shape. Intraspinal MCS with dural attachment has a more favorable prognosis compared to that derived from other tissues, because early diagnosis and surgical intervention can be managed due to compression of the spinal cord or nerve roots [10]. Although most of intraspinal MCS showed dural attachment in surgery, the dural tail sign was much rarer observed on enhanced MR images. Saito reviewed 19 intradural MCS from the literature, 12 showed dural attachment and only two had the dural tail sign [11]. It seems to be difficult to distinguish MCS from spinal meningioma if the dural tail sign is also present on enhanced MRI. MCS usually has findings of chondroid-type rings-and-arcs pattern of calcification as well as solitary or punctate calcification similar to the current case [8]; while calcified meningioma is uncommon and accounts for only 1–5% of all spinal meningiomas. Furthermore, most of calcified spinal meningioma present as calcification of the entire mass or punctuate calcification at the central portion of the mass [12]. Therefore, peripheral punctate calcification in intraspinal extramedullary tumors is supposed to be an important feature suggesting MCS.

The accurate diagnosis of intraspinal MCS relies on histopathologic examination. Under light microscope, the tumor has a bi-directional differential feature which consists of undifferentiated small-round mesenchymal cells and islands of hyaline cartilage. Immunohistochemistry of biomarkers such as Vimentin, S-100, and FLI-1 may help us to differentiate some of other tumors. From the perspective of molecular pathology, the novel HEY1-NCOA2 fusion had been discovered in 2012 and appeared to be the defining and diagnostic gene fusion in MCS [13].

Despite a well-defined appearance, intraspinal MCS has a tendency to metastasize with a high recrudescence rate and should be intervened actively. Surgery is the best treatment for intraspinal MCS. However, the effects of additional both chemotherapy and radiotherapy have not been fully clear in clinic and there is no general agreement on the necessity of adjuvant radiotherapy or chemotherapy in the treatment of MCS [14]. In the present case, the patient did not receive any adjuvant therapy, considering the total tumor resection and the absence of distant metastases. But close follow-up after operation was needed. Among the seven previously reported cases, one case underwent only surgical resection, and the others underwent adjuvant therapy following surgical resection. The follow-up period ranged from 5 months to 60 months, and two deaths due to brain metastasis were reported. In a 2015 retrospective study, Frezza AM et al. [15] presented a group of 113 patients of MCS, the median overall survival was 17 years, and the 5-year and 10-year estimated survival rates were 70 and 54%, respectively. Intraspinal MCS was reported to have a better prognosis compared with MCS in other locations [7]. In general, because the local or distant metastases may appear 20 or more years after the appearance of the primary tumor, long-term follow-up is mandatory.

In conclusions, CT and MRI play an important role in differential diagnosis for intraspinal MCS. MCS should be added to the differential diagnosis of intraspinal dumbbell-shaped tumors, especially when radiological examinations reveal punctate calcification in a homogeneous enhanced tumor without dural tail sign. However, the final diagnosis depends on histopathological results. Despite the good prognosis of intraspinal MCS, close follow-up after operation is still necessary.

## Data Availability

The datasets used and/or analysed during the current study are available from the corresponding author on reasonable request.

## Abbreviations

MCS: Mesenchymal chondrosarcoma
T1WI: T1-weighted images
T2WI: T2-weighted images
